# Cost and healthcare utilization of methicillin-resistant *Staphylococcus aureus* bacteremia estimated from linked antimicrobial resistance surveillance and hospital claims data in Japan

**DOI:** 10.1017/ash.2022.280

**Published:** 2022-08-30

**Authors:** Tomokazu Shoji, Ryusei Muto, Haruhisa Fukuda, Yuichi Muraki, Keishi Kawata, Manabu Akazawa

**Affiliations:** 1 Department of Public Health and Epidemiology, Meiji Pharmaceutical University, Tokyo, Japan; 2 Department of Pharmacy, University of Yamanashi Hospital, Yamanashi, Japan; 3 Department of Health Care Administration and Management, Graduate School of Medical Sciences Kyushu University, Fukuoka, Japan; 4 Department of Clinical Pharmacoepidemiology, Kyoto Pharmaceutical University, Kyoto Japan

## Abstract

**Objective:**

To compare the incremental costs and healthcare utilization of methicillin-resistant *Staphylococcus aureus* (MRSA) bacteremia with those of methicillin-susceptible *S. aureus* (MSSA) bacteremia.

**Design:**

Retrospective cohort study using data from April 2014 to March 2015.

**Setting:**

Antimicrobial resistance surveillance and hospital claims data from 16 Japanese hospitals.

**Patients:**

The study included 73 patients with *S. aureus* bacteremia: 23 with MRSA and 50 with MSSA.

**Methods:**

MRSA bacteremia was identified using blood cultures and drug-susceptibility tests. MRSA- and MSSA-related medical practices were evaluated. The costs were calculated and compared. All the medical costs were classified into empirical and definitive therapy periods and expressed in Japanese yen (JPY, 1 USD = 106 JPY). Additionally, costs at aggressive and passive bacterial test-performing facilities were compared.

**Results:**

No significant differences existed in MRSA-related resource use per patient episode between MRSA and MSSA bacteremia during empirical therapy. However, during definitive therapy, in MRSA bacteremia compared with MSSA bacteremia, this difference was higher. The average MRSA-related costs of empirical therapy for MRSA and MSSA were 13,380 and 9,140 JPY (126 and 86 USD) per patient, and for definitive therapy, they were 69,810 and 29,510 JPY (659 and 278 USD) per patient, respectively. No significant differences were noted. Conversely, the average examination costs during definitive therapy differed significantly: 9,740 vs 3,850 JPY (92 vs 36 USD), respectively (*P =* .0294). Furthermore, the incremental costs in aggressive facilities were lower for the definitive therapy period than those in passive facilities.

**Conclusions:**

In the definitive therapy period, MRSA bacteremia had higher incremental costs and greater use of healthcare resources. In addition, the incremental costs in aggressive facilities were lower than those in passive facilities.

Antimicrobial-resistant (AMR) infections are a serious global threat.^1^ Methicillin-resistant *Staphylococcus aureus* (MRSA) is the most common AMR infection among community-acquired and nosocomial infections. *S. aureus* bacteremia is a serious clinical disease with an annual incidence of 3.6−38.2 per 100,000 person years in the United States and a mortality rate of 20%−40%.^2,3^ Moreover, MRSA bacteremia is associated with a significantly higher mortality rate than methicillin-susceptible *Staphylococcus aureus* (MSSA) bacteremia.^4^ In Japan in 2017, there were ∼4,224 (95% confidence interval [CI], 2,769–5,994) MRSA bacteremia deaths.^5^

Worldwide, MRSA bacteremia is associated with high morbidity and mortality rates and is considered an economic burden.^6–8^ A previous study reported that, compared with patients without MRSA bacteremia, the additional medical cost per patient with MRSA bacteremia was ∼1.9 million Japanese yen (JPY, 1 USD = 106 JPY).^9^ Additionally, patients with MRSA bacteremia have higher healthcare costs than those with MSSA.^4,10,11^ Another study reported that, for the inpatient cost for Japanese hospitals, the adjusted effects were 1.04-fold (95% CI, 1.01–1.06) higher in the MRSA group (n = 7,188 patients) than in the MSSA group (n = 7,717 patients).^12^

However, further controversy has ensued regarding the burden of MRSA bacteremia. First, several previous studies have failed to provide bacterial information, leading to selection bias. Because patients who were administered anti-MRSA drugs for >3 days were considered to have MRSA bacteremia, inclusion of non-MRSA bacteremia (eg, *Enterococcus faecium*) patients may have resulted in an overestimation of the costs. Second, previous reports have included the medical care costs of empirical therapy before identifying MRSA bacteremia; therefore, MRSA-related costs may have been under- or overestimated. Blood samples are collected frequently from patients with symptoms of infections such as fever, leukocytosis, or leukopenia.^13,14^ However, there may have been a delay in detecting the bacterial species following the sample collection, or the causative organism may have been unidentified. Empirical therapy is defined as the preferred treatment when microbial pathogen identification and susceptibility testing are unavailable, whereas definitive therapy is the treatment selected following pathogen identification and susceptibility testing.^15^ Therefore, empirical therapy should comprise treatments that target the suspected bacterial species. Third, some studies may have overestimated the medical care costs by including all medical costs rather than only infection-related costs, for instance, by including the costs of surgical procedures associated with underlying diseases unrelated to the infections.

Furthermore, in a previous study, we conducted a principal component analysis–based cluster analysis using administrative claims and antimicrobial susceptibility data from 124 hospitals in Japan.^16^ The 124 hospitals were classified into 5 clusters. The characteristics in terms of infection control and risk factor of bacterial resistance were (1) aggressive performance, moderate risk, (2) moderate performance, high risk, (3) moderate performance, moderate risk, (4) passive performances, low risk, and (5) passive performance, high risk. The characteristics of the facility’s performance in bacterial testing and the rate of surgeries were found to be important for MRSA detection. Therefore, in this study, we hypothesized that the degree of examination is associated with MRSA-related economic burden.

Hospital claims could facilitate estimating the economic burden of AMR infections. However, data related to AMR infections are frequently compromised by a lack of clinical data, including microbiological data. Therefore, combining microbiological and hospital claims data may improve the reliability of findings. The Department of Medical Management at the Kyushu University Graduate School of Medicine managed the project. Microbiological and hospital claim data were collected from voluntary hospitals to analyze and clarify the type of infectious disease treatment used among hospitalized patients and the effect on medical costs.^17^ Using this database, we estimated the economic burden of MRSA bacteremia, addressed the limitations of our previous study, and analyzed facility-related infection control characteristics.

## Methods

### Data source

The hospital claims data were based on the diagnostic procedure combination per-diem payment system, which is the national administrative claims data system for acute inpatient cases in Japan. Nationally, uniform electronic diagnostic procedure combination data includes facility information (eg, number of beds), patient clinical information (eg, age, sex, disease, the *International Classification of Disease, Tenth Revision* code, and admission and discharge dates), and medical procedure information (eg, drug administration, surgery, examination, procedure, medical codes, and cost). The Japan Nosocomial Infections Surveillance (JANIS) data, which includes microbiological data, were collected by the Ministry of Health, Labor, and Welfare to enhance the availability of information on the epidemiology of nosocomial infections in Japanese hospitals. These data provide detailed bacterial information such as specimen reception date, specimen source, bacteria type, and susceptibility test results.

Both the data sets (provided voluntarily by the hospitals) had common patients and facilities. Therefore, both the hospital claims data and JANIS formats were merged according to patient identification for the patient-level analysis.

### Inclusion and exclusion criteria

This study included patients (1) who were admitted and discharged between April 1, 2014 and March 31, 2015; (2) with a positive *S. aureus* culture from the first blood cultures or after day 3 of hospitalization; and (3) who were aged ≥18 years. We excluded those (1) who were missing medical practices data; (2) with positive *S. aureus* blood cultures on day 1 or 2 of hospitalization (community-acquired infections); (3) with a history of MRSA isolated from nonblood specimens; and (4) with a history of bacteremia resulting from a previous stay in the hospital during the study period.

### Patient characteristics and medical practice

The following baseline patient demographics were collected on admission: age, sex, the Charlson comorbidity index, comorbidities, and the allocated facility cluster type. The patient characteristics were collected before the first blood-culture collection: days from admission to collection of the blood culture, admission to the intensive care unit, surgery, central venous catheter, drain, blood transfusion, chemotherapy, immunosuppressants, radiotherapy, hemodialysis, granulocyte colony-stimulating factor, and prescribed antimicrobials. The detailed definitions are shown in Supplementary Table 1.

### Variable definitions

#### Definition of antimicrobial susceptibility testing data

*S. aureus* resistance was determined using drug-susceptibility tests with oxacillin or cefoxitin, according to the 2012 Clinical and Laboratory Standards Institute guidelines. All specimens (ie, blood, urine, respiratory, and any other available) included in the JANIS database were analyzed.

#### Definition of the study period

The observation period lasted from the culture date to 42 days later, which is considered the treatment duration for complicated MRSA bacteremia.^18^ Moreover, the observation period was classified alternatively as being either 7 days long (days 1–3, 4–7, 8–14, 15–21, 22–28, 29–35, and 36–42) or 2 days long (days 1–3 and days 4–42). The empirical therapy period was defined as the period from the submission date of the bacterial test to 3 days later and the definitive therapy period thereafter. The observation was discontinued immediately when a patient was discharged or died during the observation period.

#### Definition and calculation of MRSA-related medical practices

Medical practices were classified according to the Japanese medical fee points. The MRSA-related medical practices comprised the following steps: (1) patients were selected based on the inclusion and exclusion criteria; (2) patients were divided into groups after MRSA or MSSA bacteremia was diagnosed from the blood culture; (3) all the medical procedures performed on patients in MRSA group were divided into 2 types (before and after MRSA detection); and (4) we defined and categorized MRSA-related medical practices, which involved more than double the number of patients on and after MRSA detection than before MRSA detection. Because this involved costs directly related to infection treatment, the basic hospitalization charge (hospital charge) and the items calculated at the time of admission and discharge were excluded. Antibacterial drugs classified as “J01” by the World Health Organization Anatomical Therapeutic Chemical Classification System were considered MRSA-related medical practices.

The numbers of MRSA-related medical practices were expressed as the mean time per patient with standard deviation (SD). Antibiotics for injection were analyzed using the length of therapy, defined as the number of calendar days that a patient received ≥1 dose of antibiotic, regardless of the number of agents administered during that period. Additionally, we calculated the percentage of patients who were prescribed injectable antibiotics for each antibiotic for the 2-day divided observation period.

### Cost calculations for MRSA-related and all medical practices

MRSA-related medical practices were categorized into 7-day observation periods and the average MRSA-related medical practice costs were calculated in MRSA and MSSA groups, respectively. Furthermore, to compare MRSA-related medical costs with previous estimates,^9,12^, the medical service costs for all medical practices were covered and calculated. Medical costs were also categorized based on medical fee points.

### Comparison of MRSA-related costs in MRSA and MSSA groups according to the facility cluster type

We collected and calculated 10 variables shown in the previous study for each facility and classified them into 5 clusters.^16^ Furthermore, the clusters were categorized as follows according to the bacterial test status: aggressive, passive, and moderate. The detailed facility data are shown in Supplementary Table 2. MRSA-related costs were also calculated and compared with the average cost per patient for MRSA and MSSA according to the aggressive- and passive-facility cluster types allocated to each patient.

### Statistical analysis

As indicators of the clinical and economic burden of MRSA, the outcomes included the hospital length of stay, in-hospital mortality, and medical costs for the blood cultures submitted for 42 days. Categorical variables are expressed as numbers (percentages) and continuous variables as mean (±SD) or median (interquartile ranges). The differences in patients and medical service data were analyzed using the χ^2^ test, the Fisher exact test, or the independent *t* test. Statistical significance was set at *P* < .05. In Japan, the medical service fee used to evaluate costs is determined by grading individual technologies and services. The respective cost estimates were reported in Japanese yen. All the analyses were performed using SAS version 9.4 software (SAS Institute, Cary, NC).

### Ethical considerations

This retrospective study was approved by the Ethics Committee of Meiji Pharmaceutical University Graduate School of Medicine (approval no. 202112). Due to the retrospective nature of this study, the requirement for patient informed consent was waived.

## Results

**Fig. 1.**
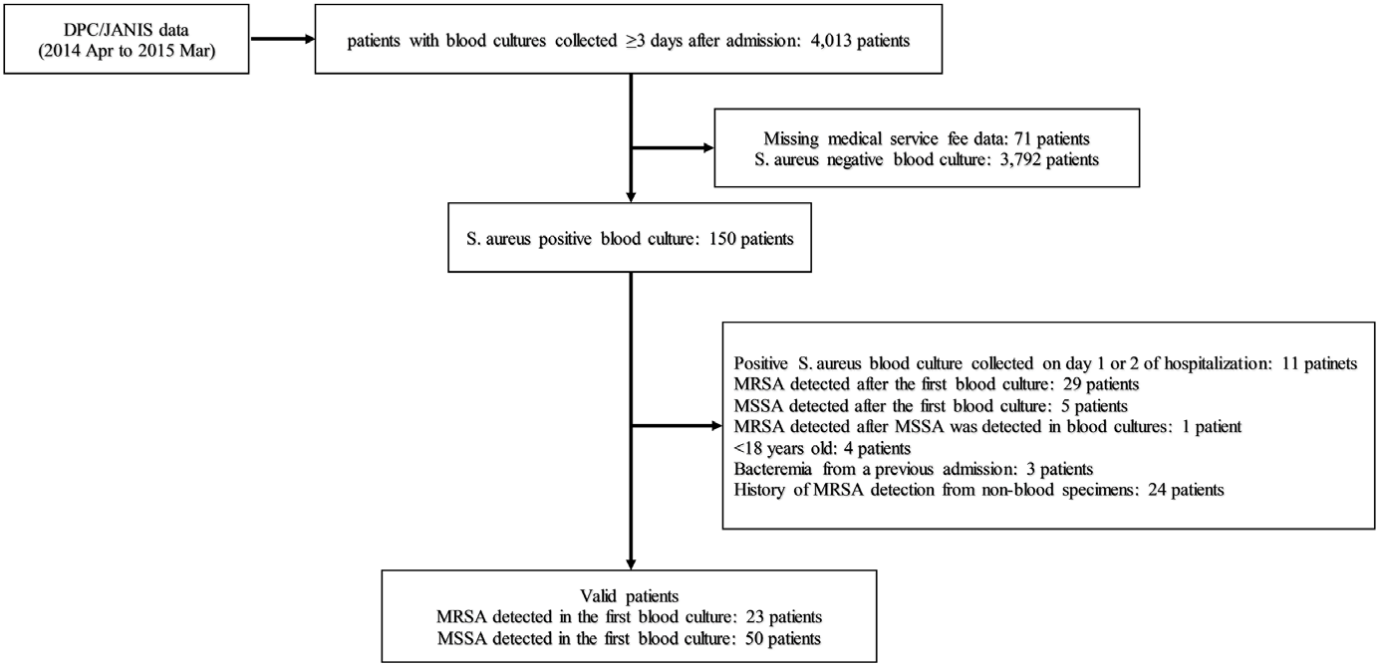
Flowchart showing the study participant selection process. Note. DPC, diagnostic procedure combination; JANIS, Japan Nosocomial Infections Surveillance; MRSA, methicillin-resistant *Staphylococcus aureus*; MSSA, methicillin-susceptible *Staphylococcus aureus*; *S. aureus*, *Staphylococcus aureus*.

During the study period, 4,013 patients had blood cultures collected ≥3 days after admission. *S. aureus* was detected in the first blood cultures of 73 valid patients (23 with MRSA [31.5%] and 50 with MSSA [68.5%]) from the 13 of 16 hospitals included in this study (Fig. 1). The mean patient age was 71.8 years (SD, ±18.6) and 72.2 years (SD, ±13.8) in MRSA and MSSA groups, respectively (Table 1). Comorbidities at admission did not differ between the 2 groups. Furthermore, 82.6% of the patients in the MRSA group and 54.0% of those in the MSSA group were prescribed antimicrobials before the culture date. After the index date, the mean lengths of stay were 25.5 days and 24.5 days in MRSA and MSSA groups, respectively (*P =* .8615). Also, 13 patients remained hospitalized until the 42-day follow-up period: 6 patients (26.1%) in the MRSA group and 7 patients (14%) in the MSSA group. The in-hospital mortality rates after blood-culture collection until the 42-day follow-up period were 30.4% and 22.0% in MRSA and MSSA groups, respectively (*P =* .4373).

**Table 1.** The Demographic Characteristics of the Patients With MRSA and MSSA Bacteremia

Characteristics	MRSA Cases (n=23), No. (%)^a^	MSSA Cases (n=50), No. (%)^a^	*P* Value
Age, mean y (SD)	71.8 (18.6)	72.2 (13.8)	.9164
Sex, male	15 (65.2)	32 (64.0)	.9196
Charlson comorbidity index, mean (SD)	1.9 (2.5)	1.4 (2.0)	.3886
**Comorbidities at hospital admission**			
Immune dysfunction	0 (0)	2 (2.7)	1.0000
Cirrhosis	3 (13.0)	4 (8.0)	.6710
Diabetes	6 (26.1)	15 (30.0)	.7315
Chronic heart failure	8 (34.8)	12 (24.0)	.3373
Chronic obstructive pulmonary disease	2 (8.7)	0 (0)	.0963
**Medical practices before the blood culture**			
Days from admission to collection of the blood culture^a^	20.3 (21.3)	17 (16.3)	.4647
ICU admission	2 (8.7)	0 (0)	.0963
Surgery	6 (26.1)	12 (24.0)	.8476
Central venous catheter	6 (26.1)	21 (42.0)	.1908
Drain	4 (17.4)	4 (8.0)	.2509
Blood transfusion	4 (17.4)	13 (26.0)	.4189
Chemotherapy	2 (8.7)	6 (12.0)	1.0000
Immunosuppressants	3 (13.0)	10 (20.0)	.7428
Radiotherapy	1 (4.4)	3 (6.0)	.4142
Hemodialysis	0 (0)	6 (12.0)	.1675
G-CSF	0 (0)	2 (4.0)	1.0000
Prescribed antimicrobial agents	19 (82.6)	27 (54.0)	.0187
**Facility cluster type**			
Aggressive facility cluster	13 (56.5)	27 (54.0)	
Passive facility cluster	3 (13.0)	3 (6.0)	
Moderate facility cluster	7 (30.4)	20 (40.0)	

Note. COPD, chronic obstructive pulmonary disease; G-CSF, granulocyte colony stimulating factor; ICU, intensive care unit; MRSA, methicillin-resistant *Staphylococcus aureus*; MSSA, methicillin-susceptible *Staphylococcus aureus*.

a Units unless otherwise specified.

**Fig. 2.**
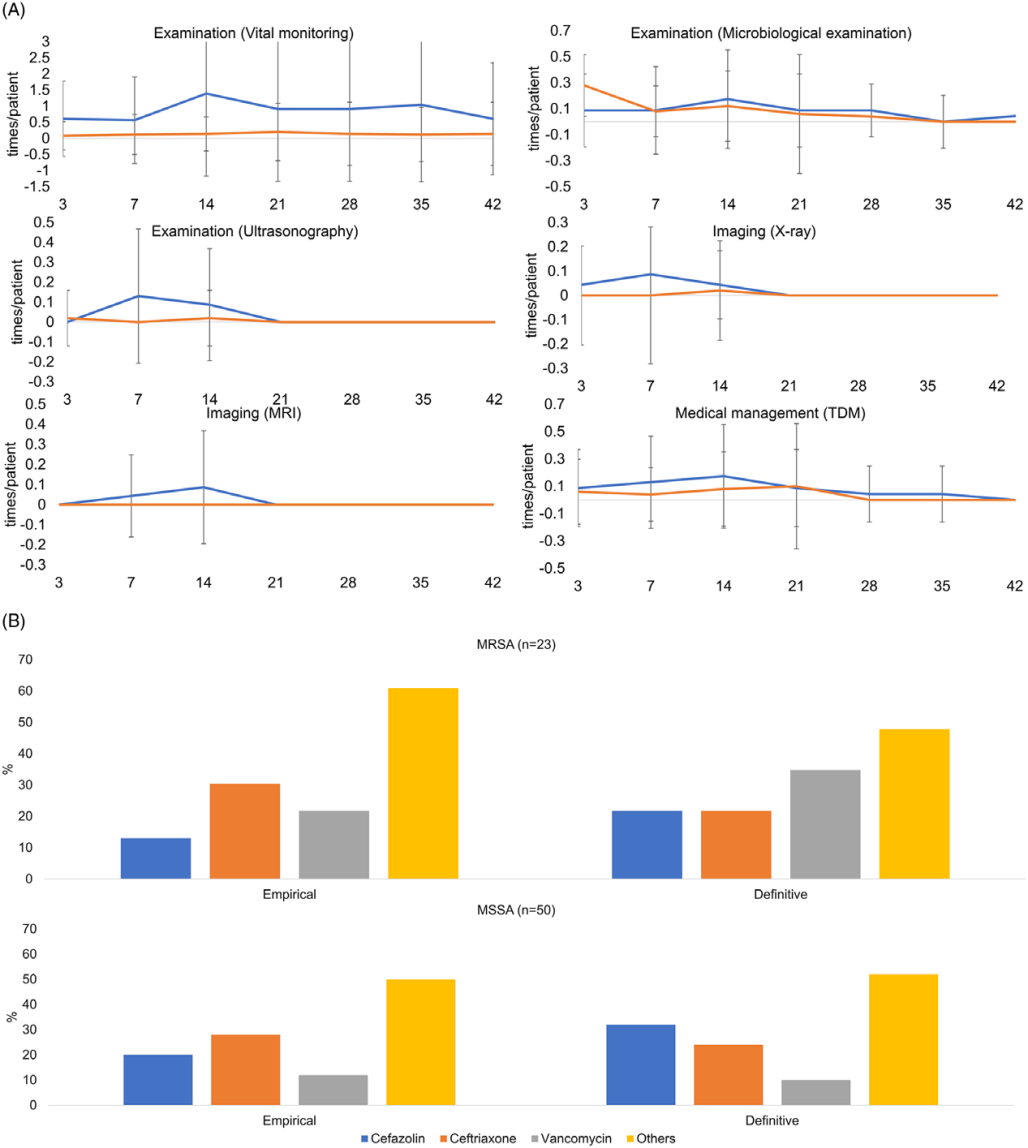
(A) The MRSA-related medical practices performed in MRSA and MSSA groups. The 7-day observation period (days 3, 7, 14, 21, 28, 35, and 42) began on day 3 because the period prior to the confirmed positive bacterial test was considered as the empirical therapy period. Note. X-axis: 7-day divided observation period after the index date. Y-axis: the mean frequency with which medical prices were performed (times/patient). Error bar: the standard deviation of the mean for each Y-axis value. Blue line: MRSA group. Red line: MSSA group. MRI, magnetic resonance imaging; TDM, therapeutic drug monitoring. (B) Percentage of patients who were prescribed antibiotics in MRSA and MSSA groups after the culture date. The empirical therapy period was the period before the bacterial identification (days 1–3), the definitive therapy period was the period after the bacterial identification (days 4–42). Note. Vertical bars: percentage of patients. X-axis: type of antibiotics. Upper graph: MRSA group. Lower graph: MSSA group. MRSA, methicillin-resistant *Staphylococcus aureus*; MSSA, methicillin-susceptible *Staphylococcus aureus*.

In this study, MRSA-related medical practices comprised 8 categories: examination, imaging, injection, medical management, procedure, surgery, injection drug, and oral drug (Supplementary Table 3). Figure 2A shows the MRSA-related medical practices performed in MRSA and MSSA groups. During the empirical therapy period, vital monitoring, microbiological examination, ultrasonography, radiography, magnetic resonance imaging, and therapeutic drug monitoring were performed with a similar frequency in the MRSA and MSSA groups. In contrast, these medical practices were performed more frequently in the MRSA group during the definitive therapy period. The median lengths of therapy for antibiotics were 11 days (interquartile range [IQR], 7–18) and 7 days (IQR, 4–14) for MRSA and the MSSA groups, respectively. Figure 2B shows the percentage of antimicrobial-prescribed patients during the 2-day observation period for the MRSA and MSSA groups. During the empirical therapy period, the most frequently used antibiotic was ceftriaxone (30% and 28% in the MRSA and MSSA groups, respectively). During the definitive therapy period, the most frequently used antibiotics were vancomycin in the MRSA group (34%) and cefazolin in the MSSA group (32%).

**Fig. 3.**
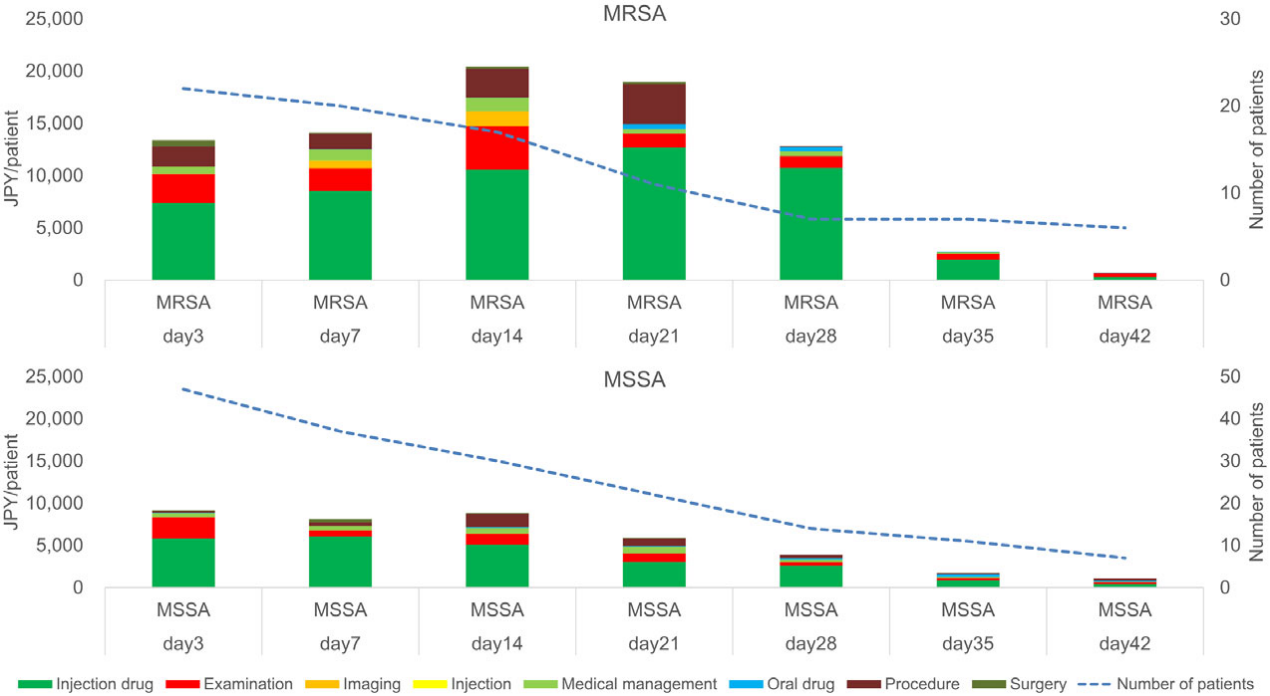
Comparison of MRSA-related medical costs in MRSA and MSSA groups. The 7-day divided observation periods (days 3, 7, 14, 21, 28, 35, and 42) began on day 3 because the period prior to the confirmed positive bacterial test was considered as the empirical therapy period. Note. Vertical bars: cost/medical practice. X-axis: 7-day divided observation period after the index date. Upper graph: MRSA group. Lower graph: MSSA group. JPY, Japanese yen; MRSA, methicillin-resistant *Staphylococcus aureus*; MSSA, methicillin-susceptible *Staphylococcus aureus*.

The mean MRSA-related costs for 42 days in MRSA and MSSA groups were 83,200 (117,710) and 38,660 (65,220) JPY per patient, respectively. Therefore, the estimated incremental cost difference between the groups was 44,540 JPY per patient, with higher costs in the MRSA group. The MRSA-related medical costs per category during both the empirical and definitive therapy periods are shown in Figure 3. The average MRSA-related costs in MRSA and MSSA groups during the empirical therapy period were 12,370 and 9,140 JPY per patient, respectively. In the definitive therapy period, these costs were 69,810 JPY per patient in the MRSA group (P = .0579), and 29,510 JPY per patient in the MSSA group (P = .1266) We detected significant differences in the average examination costs in the MRSA and MSSA groups during the definitive therapy period:9,740 vs 3,850 JPY per patient (P = .0294).

**Fig. 4.**
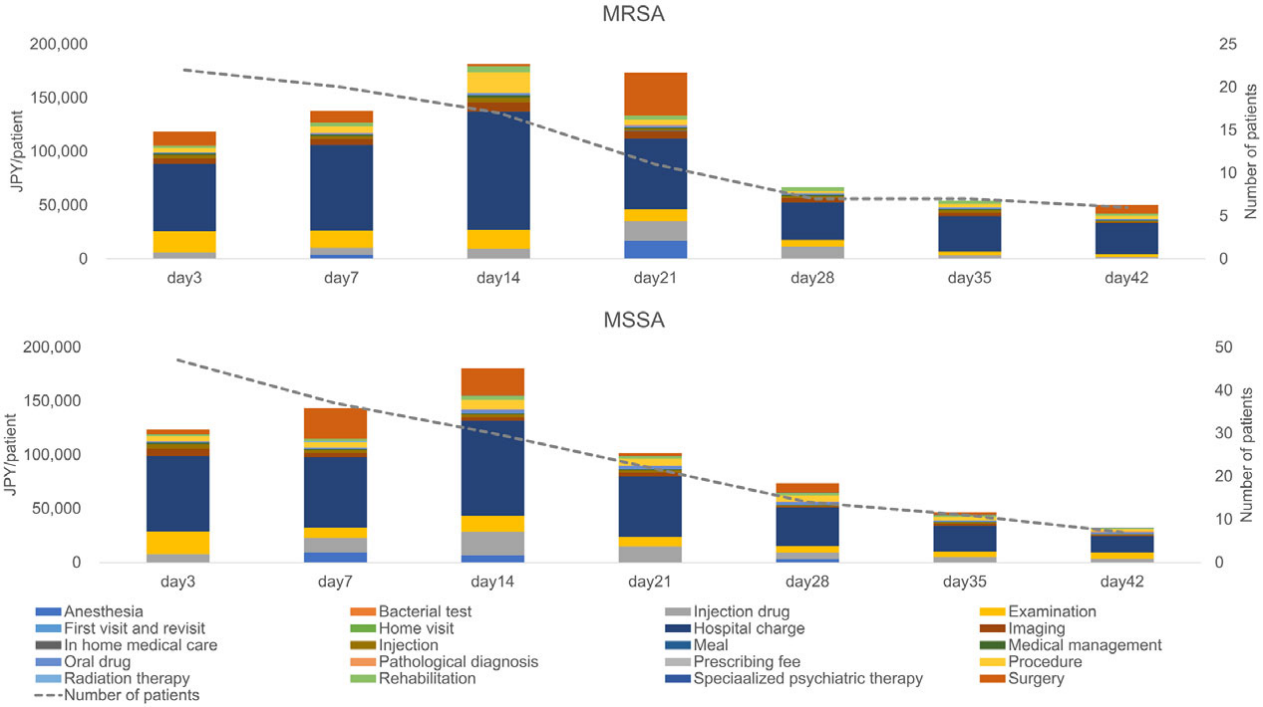
Comparison of all the medical costs in MRSA and MSSA groups. The 7-day divided observation periods (days 3, 7, 14, 21, 28, 35, and 42) began on day 3, since the period prior to the confirmed positive bacterial test was considered as the empirical therapy period. Note. Vertical bars: cost/medical practice. X-axis: 7-day divided observation period after the index date. Upper graph: MRSA group. Lower graph: MSSA group. JPY, Japanese yen; MRSA, methicillin-resistant *Staphylococcus aureus*; MSSA, methicillin-susceptible *Staphylococcus aureus*.

The average total medical costs for 42 days in MRSA and MSSA groups were 937,140 and 863,850 JPY (8,841 and 8,150 USD) per patient, respectively (Fig. 4), amounting to an additional 73,290 JPY (691 USD) per patient in the MRSA group. The estimated cost difference between all medical practices and MRSA-related medical practices was 28,750 JPY (271 USD) per patient. In MRSA and MSSA groups, respectively, the hospital charges comprised 53.5% and 52.3% of the totals; surgical costs comprised 8.8% and 9.5%; and anesthesia costs comprised 2.5% and 2.5%.

**Fig. 5.**
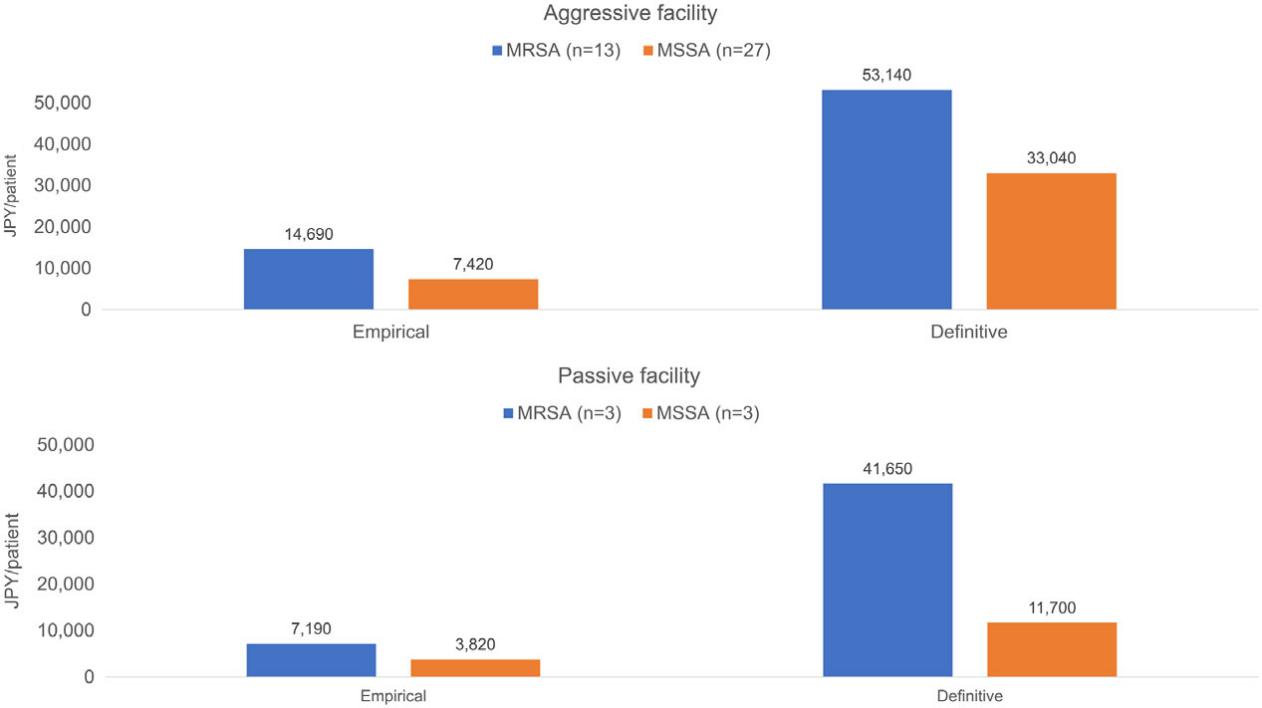
Comparison of MRSA-related medical costs per facility type. The empirical therapy period was the period before the bacterial identification (days 1–3), the definitive therapy period was the period after the bacterial identification (days 4–42). The distribution of the average medical cost/therapy period (empirical or definitive). Note. Vertical bars: cost/therapy period. Blue bars: MRSA group. Red bars: MSSA group. MRSA, methicillin-resistant *Staphylococcus aureus*; MSSA, methicillin-susceptible *Staphylococcus aureus*.

The numbers of facilities in each facility cluster type were as follows: aggressive (n = 7), passive (n = 2), moderate (n = 4). Figure 5 shows the MRSA-related cost differences between MRSA and MSSA groups for the empirical and definitive therapy periods per facility cluster type. In the aggressive facility cluster, relative to the MSSA group, the MRSA group incurred incremental costs during both the empirical and definitive therapy periods by ∼7,270 JPY (69 USD) per patient and 20,100 JPY (190 USD) per patient, respectively. Similarly, in the passive facility cluster, relative to the MSSA group, the MRSA group incurred incremental costs during both the empirical and definitive therapy periods by ∼3,370 JPY (32 USD) per patient and 29,950 JPY (283 USD) per patient, respectively.

## Discussion

We estimated the MRSA-related medical costs that considered the detected bacterial data, distinguished between the empirical and definitive therapy periods, and focused on MRSA-related medical practices. These results suggest that MRSA-related economic burden is greater than that of MSSA; however, previous studies that included all medical costs may have overestimated the MRSA-related medical costs. When analyzing the facility-related infection control characteristics, the aggressive-facility cluster incurred lower incremental costs during the definitive therapy period than the passive-facility cluster. In other words, facilities that actively performed bacterial tests may have a system for prompt reporting of bacterial test results. This may eliminate the need for various medical procedures in the definitive therapy in which cultures are submitted for testing, thereby enabling the implementation of appropriate treatment at an earlier stage. Therefore, the use of aggressive bacterial testing may lead to decreased incremental costs of MRSA bacteremia.

Tests were performed more frequently in the MRSA group during the definitive therapy period. Specifically, examinations focused on locating disseminated lesions such as vital monitoring, radiography, and ultrasonography. Similarly, Rubio et al^19^ reported that the resource use per bacteremia episode (eg, radiographs and ultrasound scans) was higher for MRSA cases than for MSSA cases. The frequency of medical examinations may have increased because of the need for additional tests due to the severity of MRSA bacteremia.

We hypothesized that similar antimicrobial therapy was provided in both MRSA and MSSA groups during the empirical period. Moreover, we hypothesized that there would be cost differences during the definitive period because the optimal treatment was bacteria dependent. Here, ceftriaxone use was the highest during the empirical therapy both in MRSA and MSSA groups, and during the definitive therapy period, cefazolin use was the highest in the MSSA group, and anti-MRSA drug use was the highest in the MRSA group. As in previous research, antimicrobial agents targeting both gram-positive cocci and gram-negative bacilli were administered during the empirical therapy period.^20^ During the definitive therapy period, anti-MRSA drugs used, such as vancomycin, were more expensive than MSSA treatment antibiotics, potentially contributing to the economic burden of MRSA infections.

For all medical practices, the average of all medical costs for 42 days was higher in the MRSA group than in the MSSA group. The estimated cost difference between all medical practices and MRSA-related medical practices was 28,750 JPY (271 USD) per patient. This difference pertained to hospital charges, surgeries, the accompanying drug use, and anesthesia. A previous study reported that the hospital charges of patients with MRSA and MSSA were 15,000 USD and 14,000 USD, respectively.^12^ However, the costs unrelated to *S. aureus* infections were also included. Our results suggest that previous reports may have overestimated these costs.

The frequency of infection control examinations may influence the prevalence of AMR infections. We hypothesized that aggressive infection control facilities incurred higher costs during the empirical therapy period, but these costs stabilized after detecting the specific bacteria. In contrast, passive infection-control facilities with low activity during the empirical period may incur higher costs during the definitive therapy period due to additional medical practice utilization. These findings suggest that the incremental costs incurred by aggressive facilities during the definitive therapy period may be lower than those incurred by passive facilities. *S. aureus* bacteremia requires appropriate follow-up, including bacterial and imaging tests, and early implementation of these could lead to cost saving during the targeted treatment period.^21^

This study had several limitations. First, the JANIS data did not include accurate infection onset dates. Although the culture collection dates were recorded, the JANIS data did not indicate when the bacteria were detected. Here, we considered a positive *S. aureus* culture from day 3 onward. Second, the facility-related infection control characteristics may depend on the study period. In this study, the study period may have affected the cluster classification.

We did not detect significant differences in the overall costs between the empirical and definitive therapy periods. In contrast, there were significant cost differences for examinations between the MRSA and MSSA groups during the definitive period. Moreover, the use of aggressive bacterial test practices may have decreased the incremental costs of MRSA bacteremia during the definitive therapy period. The information on identified bacterial cultures will lead to more detailed research in the future.

## Supplementary material

For supplementary material accompanying this paper visit https://doi.org/10.1017/ash.2022.280. click here to view supplementary material
