# Supplementary material for: Cost and healthcare utilization of methicillin-resistant Staphylococcus aureus bacteremia estimated from linked antimicrobial resistance surveillance and hospital claims data in Japan
Source: Antimicrob Steward Healthc Epidemiol. 2022 Aug 30;2(1):e147. doi: 10.1017/ash.2022.280 (PMC9726553; doi:10.1017/ash.2022.280)
Supplement: Supplementary file 1 [file S2732494X22002807sup001.docx]

**Supplemental material**

**Supplementary Table 1:** Patient variable definitions.

| Characteristics | Definition |
| --- | --- |
| **Patient information collected at the time of admission** |  |
| Age | Age at the time of admission to hospital |
| Sex | Male or female |
| Charlson Comorbidity Index | Comorbidity index on admission to hospital |
|  |  |
| Immune dysfunction | ICD-10: (D70-D89) |
| Cirrhosis | ICD-10: (B18, K70-K71, K74, K76, K78 P78) |
| Diabetes | ICD-10:(E10-E14) |
| Chronic heart failure | ICD-10: (I50) |
| COPD | ICD-10: (J44) |
|  |  |
| **Medical practices before the blood culture** |  |
| Days from admission to collection of the blood culture | Days from admission to the culture date |
| ICU admission | Admission to the intensive care unit |
| Surgery | Whether or not the surgery code was recorded |
| Central venous catheter | With or without central venous catheter insertion |
| Drain | With or without to use drains |
| Blood transfusion | With or without blood transfusion |
| Chemotherapy | Whether or not chemotherapy was administered |
| Immunosuppressants | Whether or not immunosuppressive drugs were administered |
| Radiotherapy | With or without radiation therapy |
| Hemodialysis | Whether or not hemodialysis was performed |
| G-CSF | Whether or not G-CSF drugs were administered |
| Prescribed antimicrobial agents | Whether or not antimicrobials were administered |
| Facility cluster type | Categorized aggressive or passive or moderate cluster type |

COPD, Chronic obstructive pulmonary disease; G-CSF, Granulocyte colony stimulating factor; ICD, International statistical classification of diseases and related health problems; ICU, Intensive care unit.

**Supplementary Table 2:** Demographic characteristics per facility cluster.

| Characteristics |  |  |  |  |  |  |  |  |  |  |  |  |  |  |  |  |
| --- | --- | --- | --- | --- | --- | --- | --- | --- | --- | --- | --- | --- | --- | --- | --- | --- |
| Hospital identification | 1 | 2 | 3 | 4 | 5 | 6 | 7 | 8 | 9 | 10 | 11 | 12 | 13 | 14 | 15 | 16 |
| Type of clusters | A | A | A | A | A | A | A | B | B | B | B | C | C | C | D | E |
| Patient number/annum | 12,493 | 12,808 | 11,190 | 5,864 | 13,035 | 4,038 | 2,957 | 16,347 | 3,295 | 1,552 | 3,249 | 7,936 | 3,655 | 5,393 | 1,672 | 2,643 |
| Average inpatient hospital days | 14.7 | 10.8 | 10.7 | 10.5 | 11.7 | 9.8 | 15.0 | 11.1 | 16.0 | 19.3 | 12.6 | 12.7 | 14.3 | 12.7 | 12.5 | 18.2 |
| TDM for vancomycin (%) | 73.7 | 84.5 | 76.4 | 84.8 | 81.4 | 65.7 | 78.4 | 81.4 | 74.1 | 47.1 | 47.4 | 31.0 | 59.1 | 67.9 | 60.0 | 41.2 |
| Multiple blood cultures sets (%) | 72.6 | 60.6 | 11.2 | 19.3 | 85.3 | 92.9 | 87.0 | 80.6 | 77.8 | 73.7 | 84.3 | 37.0 | 53.9 | 85.5 | 85.5 | 11.8 |
| Blood culture collected prior to broad spectrum antibiotic therapy (%) | 55.2 | 58.3 | 48.6 | 74.8 | 78.6 | 74.9 | 68.4 | 64.2 | 29.5 | 56.1 | 38.1 | 20.5 | 16.3 | 41.9 | 43.0 | 23.5 |
| Specimens for culture collected prior to broad spectrum antibiotic therapy (%) | 75.5 | 80.3 | 72.5 | 88.7 | 89.3 | 81.5 | 92.9 | 84.6 | 62.1 | 79.6 | 75.7 | 61.0 | 60.2 | 79.5 | 74.7 | 67.6 |
| Number of bacterial tests (/100 bed-days) | 13.8 | 13.7 | 8.0 | 19.8 | 15.9 | 17.1 | 12.8 | 10.5 | 5.1 | 5.7 | 9.9 | 7.2 | 8.8 | 10.4 | 14.7 | 7.1 |
| Contamination of blood cultures (%) | 3.7 | 12.1 | 0 | 0 | 6.0 | 1.8 | 7.7 | 0.5 | 2.3 | 2.2 | 0 | 3.6 | 4.2 | 6.4 | 0 | 6.9 |
| Number of CD detected tests (/1,000 bed-days) | 8.3 | 8.9 | 0.3 | 6.5 | 5.7 | 11.4 | 16.8 | 4.6 | 0 | 5.4 | 6.3 | 6.3 | 5.5 | 9.2 | 3.2 | 13.0 |
| Medical fee for IPC type | type 1 | type 1 | type 1 | type 1 | type 1 | type 1 | type 1 | type 1 | type 1 | type 1 | type 1 | type 1 | type 1 | type 1 | type 2 | type 2 |
| Surgery rate (%) | 25.0 | 18.1 | 16.5 | 27.9 | 26.1 | 16.9 | 26.2 | 30.4 | 32.3 | 41.5 | 36.8 | 22.3 | 29.3 | 24.3 | 29.2 | 26.2 |
| Region | East | East | East | East | East | West | West | East | West | West | West | East | West | West | East | East |

Type of clusters: A) aggressive-performance, moderate-risk, B) moderate-performance, high-risk, C) moderate-performance, moderate-risk, D) passive-performances, low-risk, E) passive-performance, high-risk in terms of infection control and risk factor of bacterial resistance.

CD, *Clostridioides difficile*; IPC, Infection prevention and control; TDM, Therapeutic drug monitoring

**Supplementary Table 3:** MRSA-related medical practices.

| Section | Name | Code |
| --- | --- | --- |
| Examination | Monitoring vital signs | 160155610 |
|  | Microbiological examination  Immunological examination | 160058710, 160146310, 160146410  160007110, 160054910, 160055010, 160055210, 160055310, 160121210, 160124350, 160124450, 160160150, 160174950, 160177150, 160195410, 160197210 |
|  | Urine/feces inspection | 160003910, 160005170 |
|  | Ultrasonography  Respiratory and circulatory function examination | 160072370, 160165010  160069610, 160070210 |
|  | Endocrinological examination | 160035510 |
|  | Electroencephalogram examination | 160119410, 160147610 |
|  | Blood chemistry examination | 160177550 |
|  | Biochemical examination | 160023610, 160023710, 160029410, 160168450, 160192510 |
| Imaging | X-ray | 170000510 |
|  | Magnetic resonance imaging | 170033510 |
| Injection | Injection | 130000310 |
|  |  |  |
| Medical management | Therapeutic Drug Monitoring | 113000410, 113000770,113014810 |
| Procedure | General treatment | 140000710, 140009310, 140023210, 140023510, 140032310 |
| Surgery | Blood transfusion | 150001010, 150225410, 150327910, 150366570 |
